# Improving patient-centeredness of fertility care using a multifaceted approach: study protocol for a randomized controlled trial

**DOI:** 10.1186/1745-6215-13-175

**Published:** 2012-09-24

**Authors:** Aleida G Huppelschoten, Noortje TL van Duijnhoven, Rosella PMG Hermens, Chris Verhaak, Jan AM Kremer, Willianne LDM Nelen

**Affiliations:** 1 Department of Obstetrics and Gynaecology, Radboud University Nijmegen Medical Centre, PO Box 9101, Nijmegen, HB 6500, the Netherlands; 2 Scientific Institute for Quality of Healthcare (IQ Healthcare), Radboud University Nijmegen Medical Centre, PO Box 9101, Nijmegen, HB 6500, the Netherlands; 3 Department of Medical Psychology, Radboud University Nijmegen Medical Centre, PO Box 9101, Nijmegen, HB 6500, the Netherlands

**Keywords:** Patient-centeredness, Quality of life, Distress, Multifaceted approach, Determinants, Protocol

## Abstract

**Background:**

Beside traditional outcomes of safety and (cost-)effectiveness, the Institute of Medicine states patient-centeredness as an independent outcome indicator to evaluate the quality of healthcare. Providing patient-centered care is important because patients want to be heard for their ideas and concerns. Healthcare areas associated with high emotions and intensive treatment periods could especially benefit from patient-centered care. How care can become optimally improved in patient-centeredness is unknown. Therefore, we will conduct a study in the context of Dutch fertility care to determine the effects of a multifaceted approach on patient-centeredness, patients’ quality of life (QoL) and levels of distress. Our aims are to investigate the effectiveness of a multifaceted approach and to identify determinants of a change in the level of patient-centeredness, patients’ QoL and distress levels. This paper presents the study protocol.

**Methods/Design:**

In a cluster-randomized trial in 32 Dutch fertility clinics the effects of a multifaceted approach will be determined on the level of patient-centeredness (Patient-centredness Questionnaire – Infertility), patients’ QoL (FertiQoL) and levels of distress (SCREENIVF). The multifaceted approach includes audit and feedback, educational outreach visits and patient-mediated interventions. Potential determinants of a change in patient-centeredness, patients’ QoL and levels of distress will be collected by an addendum to the patients’ questionnaire and a professionals’ questionnaire. The latter includes the Organizational Culture Assessment Instrument about the clinic’s culture as a possible determinant of an increase in patient-centered care.

**Discussion:**

The study is expected to yield important new evidence about the effects of a multifaceted approach on levels of patient-centeredness, patients’ QoL and distress in fertility care. Furthermore, determinants associated with a change in these outcome measures will be studied. With knowledge of these results, patient-centered care and thus the quality of healthcare can be improved. Moreover, the results of this study could be useful for similar initiatives to improve the quality of care delivery. The results of this project are expected at the end of 2013.

**Trial registration:**

Clinicialtrials.gov NCT01481064

## Background

Would it not be great if every hospital worldwide provides consistent, high-quality medical care to all patients? Unfortunately, this is still not daily reality, which underlines the importance of research projects on the improvement of quality of care [1]. The Institute of Medicine structured the concept of ‘quality of care’ in 2001 by defining six aims around the core need for high-quality healthcare; ‘safety’, ‘effectiveness’, ‘timeliness’, ‘efficiency’, ‘equity’, and ‘patient-centeredness’ [1]. Subsequently, quality measures were developed mainly focusing on safety and effectiveness, while patient-centeredness was often neglected [1-3]. Patient-centeredness is defined as ‘care that is respectful of and responsive to individual patient preferences and needs and that is guided by patient values’ [1]. Providing patient-centered care is important, because it can build caring relationships between patients and healthcare providers [4,5], improve health outcomes [4,6,7], reduce costs [4,6,8] and increase levels of patients’ quality of life (QoL) [9].

Healthcare areas associated with high emotions and intensive treatment periods (for example, oncology or rheumatic care) could especially benefit from more patient-centered care. Fertility care is also one of these areas. In developed countries, infertility affects one in six couples who have tried to achieve pregnancy [10,11]. About 55% of them seek medical help for their problem and start with a longlasting period of fertility workup and/or treatment [12]. This period is a physical and psychological burden for the couples [13]. For example, a woman undergoing in *vitro* fertilization (IVF) treatment has to inject herself for several weeks to stimulate the production of oocytes, visit the clinic multiple times for ultrasound check-up and has to undergo transvaginal retrieval of oocytes. After fertilization of the oocytes in the laboratory with sperm, the resulting embryo is transferred to the uterus. Subsequently, the couple has to wait 2 weeks to find out whether pregnancy has occurred. If not, the couple can start a new IVF cycle. Eventually, this treatment period can take several months to even years, which underlines the impact of infertility and its treatment on patient’s QoL. This may be seen in terms of impairments in psychosocial well-being, sexual satisfaction and marital relationship [13-16]. Moreover, because of the high physical and emotional burden, about 23% of couples end treatment prematurely [17]. Given these high percentage of patients deciding to terminate treatment early, frequently as a result of high psychological and psychical impact, every clinic should optimize its care towards more patient-centered care [13,14].

In Dutch fertility care, van Empel and colleagues showed that several parts of patient-centeredness could be improved [18]. How such improvement initiatives can be undertaken most successfully is still unknown. Moreover, there are potential barriers impeding improvement initiatives. For instance, professionals in fertility care underestimate the importance of patient-centeredness and have difficulties in estimating their performance correctly [19]. Another barrier may be the organizational culture of a hospital. For instance, patients visiting hospitals that support teamwork are more satisfied with their care than patients visiting hospitals with other culture types (for example, hierarchical culture) [20-23]. Moreover, providing patient-centered care is often thought to be expensive and time consuming [24,25].

Obviously, steps need to be taken to achieve a behavioral change in professionals towards providing more patient-centered care. Because no magic bullets exist for changing healthcare providers’ behavior [26], multiple interventions based on known barriers could accomplish this behavioral change and improve patient-centered care.

We designed a study to evaluate the effects of a quality improvement strategy consisting of three different elements; that is, a multifaceted approach. We hypothesize that providing clinicians with this multifaceted approach will improve the level of patient-centeredness and thus healthcare quality. If so, this is essential in improving patients’ QoL, reducing levels of distress and percentages of patients discontinuing treatment, and eventually reducing healthcare costs.

The main aim of this study is therefore to determine the effects of a multifaceted approach on patient-centeredness, patients’ QoL and levels of distress by: investigating the *effectiveness* of a multifaceted approach for care improvement on patient-centeredness, patients’ QoL and levels of distress; identifying *determinants*, at both patient and clinic levels, of an increase in the level of patient-centeredness, an increase in patients’ QoL and a decrease in distress levels; and performing a *process evaluation* to study the feasibility of the multifaceted approach and gain insight into factors that affected the impact of the intervention.

## Methods/Design

### Setting

In the Netherlands, secondary and tertiary fertility care is provided by three different types of clinics based on the kind of treatment they offer. Initial fertility assessment, ovulation induction and intra-uterine insemination are carried out in all Dutch clinics. The intermediate Dutch clinics can also start up and monitor the IVF and intra-cytoplasmic sperm injection treatments. However, oocyte retrieval and embryo transfer has to occur in one of the 13 licensed clinics (eight university hospitals, four general hospitals, and one private clinic). Almost all Dutch fertility clinics are national health service funded. Every Dutch citizen has a basic insurance coverage, which covers treatment and medication costs for ovulation induction, intra-uterine insemination, and three cycles of IVF/intra-cytoplasmic sperm injection.

### Study population

The study will be performed in a representative Dutch infertile patient group, under treatment in one of 32 Dutch clinics. All couples that participate in this study underwent at least one cycle of medically assisted reproduction (for example, ovulation induction, intra-uterine insemination, IVF, and intra-cytoplasmic sperm injection). Both women and their partners will be invited to participate in this study individually. However, because it is still unknown whether women and partner experiences with patient-centered fertility care are associated, only the women’s data will be used to answer our main research questions. Partners’ data will be used to analyze whether gender is a determinant of patient-centered fertility care. Those couples who are pregnant while completing the questionnaire set will be excluded from all analyses, because most questions about patient-centeredness, patients’ QoL and levels of distress are confounded in this patient group [18,27,28].

### Ethical approval

The Regional Review Board for Research on Human Subjects (CMO) has received full ethical approval for this project (CMO No. 2011–034). The study is registered at clinicaltrials.gov NCT01481064.

### Study design

In a cluster-randomized trial, the effects of a multifaceted approach on the level of patient-centered fertility care, patients’ QoL and the level of distress will be identified. To include a representative patient group for baseline measurement, clinics will be asked to extract the address files of all patients who underwent medically assisted reproduction in their clinics during the past 3 months (2011) from their diagnosis treatment combination coding system. Per clinic, 25 to 75 patients will be randomly selected depending on the clinic’s size. Participation is voluntary and anonymous. The couples will receive a letter with an invitation to participate. If they are willing, they complete an online questionnaire set, accessible by a personal code. Two weeks after the initial mailing, all patients will receive a reminder. Another 3 weeks later, nonresponders will receive a reminder with their personal codes and the additional option to complete a paper version of the questionnaire [29].

Following baseline measurement, all 32 participating clinics will be randomly assigned to usual care (16 clinics) or to the multifaceted approach (16 clinics) with stratification for clinic size (large/medium/small) and IVF facilities (full licensed/intermediate/no IVF facilities).

**Figure 1:**
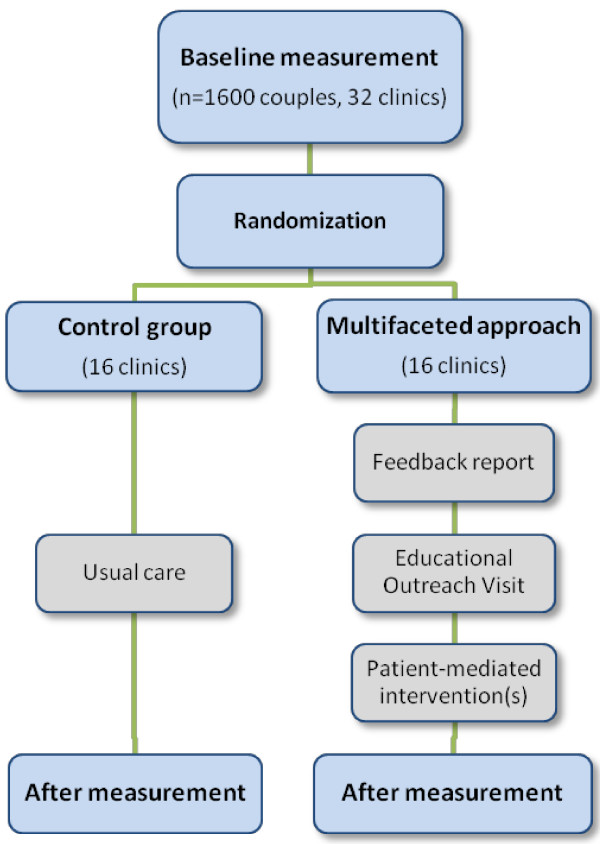
Design of the study.

After 1 year of intervention exposure, all clinics again extract the address files of all patients who underwent medically assisted reproduction in the last 3 months for the after measurement. The same questionnaire set will be used, which again have to be completed by both the women and the partners separately. Figure 1 illustrates the study design schematically.

### Questionnaires

The questionnaire set consists of three different questionnaires and some additional background questions for case-mix adjustment and to identify possible determinants of a change in the levels of patient-centeredness, patients’ QoL and distress levels.

#### Patient-centredness Questionnaire – Infertility

The Patient-centredness Questionnaire – Infertility, a validated instrument measuring patient-centeredness of fertility care by asking about patients’ experiences with care, is composed of 46 questions. This questionnaire contains seven subscales, namely: Accessibility, Information, Communication, Respect for patients’ values, Continuity and transition, Patient involvement, and Competence [18]. A higher score on the total Patient-centredness Questionnaire scale or one of the subscales represents a higher level of patient-centeredness.

#### FertiQoL questionnaire

The internationally developed and validated FertiQoL questionnaire consists of two general items and two modules measuring QoL (the FertiQoL Core and the optional FertiQoL Treatment module). The Core module involves 22 fertility-specific items covering four subscales; Mind–Body, Emotional, Relational and Social. The optional treatment module assesses QoL related to the fertility treatment itself. In this study the Dutch version of the two general items and the FertiQoL Core module will be used. A higher score on the total FertiQoL scale or one of the subscales means better QoL [27].

#### SCREENIVF questionnaire

The recently developed SCREENIVF questionnaire consists of 31 questions covering five emotional maladjustment scales (that is, five risk factors for increased emotional problems during fertility treatment); anxiety, depression, helplessness, acceptance regarding fertility problems, and perceived social support [30]. The assessments of anxiety, depression and perceived social support are based on generic instruments (that is, Spielberger State and Trait Anxiety Inventory [31,32], Beck Depression Inventory [33], and Inventory of Social Involvement [34], respectively), and the assessments of helplessness and acceptance are based on a fertility specific instrument (that is, Illness Cognition Questionnaire [35,36]). Subscale scores will be calculated according to the cutoff values described by Verhaak and colleagues [30]. Based on these five subscales, total SCREENIVF scores range from 0 to 5, indicating how many risk factors for increased emotional problems during fertility treatment are present [30].

### The intervention

Clinics randomized for the multifaceted approach will be exposed to this intervention for 1 year. The content of the multifaceted approach is based on previous interviews with Dutch gynecologists, fertility nurses and hospitals’ quality officers about their potential barriers and facilitators for quality improvement, and on previous studies on patient-centered fertility care [19,37,38]. These studies reported a large variation between clinics and the need for feedback about current performance for the clinicians involved [18,19]. However, it is shown that audit and feedback alone is not enough; the effectiveness increases if feedback is detailed, offered in high intensity, with professionals’ involvement and as part of a multifaceted intervention [39-42]. We therefore designed a multifaceted approach consisting of three elements: audit and feedback, educational outreach visits (EOVs), and patient-mediated interventions.

The feedback consists of a personalized paper report with the clinic’s own results, benchmarked and presented in relation to all 32 participating clinics. To identify aspects of care with priority for improvement, quality improvement scores will be calculated per clinic and presented in the feedback report. The higher a quality improvement score (3 – perceived experience score × importance score from the patients’ perspective), the more need there is for improvement [18]. The clinics receive this report shortly after baseline measurement and 1 month before the EOV will take place. Prior to this visit, the researcher and representative gynecologist will discuss the results from the baseline measurement and define the most important items for EOV.

During EOV, the feedback reports will be discussed with the team of each clinic exclusively paying special attention to their high quality improvement scores. The EOVs are led by a researcher involved in baseline measurement and drafting the feedback reports. For the EOV, all members of the fertility team (gynecologists, residents, nurses, secretaries, embryologists, analysts) will be invited. Each EOV results in the definition of improvement goals and a clear action plan with allocation of tasks defined by the professional team. The EOVs will also be attended by a quality officer of the hospital involved, who will manage the execution of the formulated action plan. Additionally, a representative of the Dutch Patients’ Association of Infertility ‘*Freya*’ who is a former patient of that clinic will be present. These representatives can present the needs and wishes of infertile patients during the EOV. All patients’ representatives will receive a manual about EOVs and undergo a short training program for fulfilling their role in the EOV.

Finally, to enable clinics to translate items mentioned in the feedback report to the clinic’s daily reality they are offered several patient-mediated interventions. For example, clinics can decide to organize focus groups or create online communities to gain more specific and detailed information from their patients about the care aspects with the highest quality improvement scores.

Following the EOV, the hospital’s professional team and quality officer will be mainly responsible for the execution of the action plan. However, the researchers will monitor this process carefully by contacting the team every 2 months. Additionally, all professionals and representatives of *Freya* are invited to participate in an online study community. This community will be a platform for professionals to exchange their ideas about quality improvement programs. Besides, the researcher will write a blog at least every 2 months in which the quality improvement progress of all participating clinics will be described. The ideas and progress of one clinic can stimulate another clinic to improve even more.

### Determinants of change in patient-centeredness, patients’ QoL and distress levels

#### Patient characteristics

The following patient characteristics will be collected, based on general and fertility literature as possibly being associated with patient-centered care, QoL and/or levels of distress: gender, age, ethnicity, level of education, duration of relationship, economic status, duration and cause of infertility, fertility treatments so far received, consumption of professional emotional support during fertility treatment, medical history, and recently experienced lifetime events (for example, death of a relative, being fired from work) [18,43-46].

#### Clinic characteristics

Potential determinants at the clinic level will be collected by a professionals’ questionnaire during patients’ baseline measurement and by separate data collection during the EOV. The questionnaire will be spread electronically among all healthcare professionals (for example, gynecologists, residents, nurses, laboratory employees, secretaries, and so forth) working at the fertility departments of the 32 participating hospitals. The questionnaire consists of two parts: 12 general questions about clinic characteristics (for example, number of fertility consultations per year, composition of the fertility team, mean age and sex ratio of the fertility team); and six questions from the Organizational Culture Assessment Instrument, a validated questionnaire to examine organizational culture based on the Competing Values Framework [47-49]. The Competing Values Framework recognizes that no hospital exhibits only one culture, but that multiple cultures and values coexist simultaneously [50] (that is, clan/family culture, adhocracy culture, market culture, and hierarchy culture). The four culture types relate to each other on a two-by-two matrix with two axes denoting both the flexibility and the orientation of the hospital to the outside world [47-49,51-53]. In this study the validated Dutch version of the Organizational Culture Assessment Instrument will be used [54].

Additional possible determinants at the clinic level of a change in patient-centeredness, patients’ QoL and distress levels will be collected during the EOV. According to the literature, these characteristics may influence successful implementation of the action plan – such as, for example, the level of preparation before and the enthusiasm and agreement of the professional team during the EOV [55]. The researcher will record these team characteristics on a five-point Likert scale.

### Sample size calculation

To account for a representative number of patients per clinic (that is, 25 to 75 patients per clinic) at least 1,600 couples will be included. The sample size calculation, which was based on the results of the previous Patient-centredness Questionnaire validation study [18], confirmed that this number of patients is sufficient for a proper analysis. To detect a mean difference score of 0.25 between usual care and the multifaceted approach on patient-centeredness (α = 0.05, two-sided testing, β = 0.8) at least 93 couples are required. Taking into account clustering of couples (30 couples/clinic) and a mean intracluster correlation coefficient of 0.13 [18], 1,023 couples have to be involved. With an expected response rate of 70% [18], at least 1,462 couples have to be invited at both baseline and after measurement.

### Data analysis

All data will be entered into a SPSS database (version 16.0 for Windows®; SPSS Inc., Chicago, IL, USA). Data analysis will be described following our two study aims.

#### Effectiveness of the multifaceted approach

To analyze the effectiveness of the multifaceted approach on patient-centeredness, patients’ QoL and levels of distress, the difference in baseline and after-measurement scores will be analyzed with adjustment for clustering of patients within clinics. Multilevel linear regression analyses will therefore be performed in which the intervention (multifaceted approach vs. usual care) will act as the independent variable. The Patient-centredness Questionnaire – Infertility total and subscale scores, the FertiQoL total scores and the SCREENIVF scores will be used as dependent variables. Differences at baseline will be corrected for by taking baseline scores as a covariate in the final multilevel models.

#### Determinants of change in patient-centeredness, patients’ QoL and distress levels

First, all independent variables concerning baseline patient and clinic characteristics will be checked for colinearity. These variables include all patient and clinic background characteristics, as well as the four variables concerning hospital culture, and the team characteristics collected during EOV.

If a correlation coefficient >0.6 is found between two variables, preference will be given to the variable theoretically closest to actual outpatient performance. Subsequently, all independent variables will be tested in a univariate analysis with the dependent variables concerning the differences between patient-centeredness, patients’ QoL and levels of distress in baseline and after measurement. The variables tend to be associated and show enough interclinic variation will be included in three multilevel linear regression models to explain differences in an increase in patient-centeredness, an increase in patients’ QoL and a decrease in levels of distress, respectively. To assess which part of the variation can be explained by the determinants, the explained variance (*R*^2^) per model will be calculated. Significance for all analyses will be set at *P* <0.05.

### Process evaluation

A process evaluation, according to Hulscher and colleagues [56], will be performed during and after the intervention to investigate the feasibility of the action plan formulated during the EOV. This evaluation will also make clear whether and to what extent professionals and patients used and appreciated the elements of the multifaceted approach. Especially, process evaluation is essential to find out how and to what extent clinics accomplished the third part of the multifaceted approach; that is, patient-mediated interventions.

During the intervention, telephonic interviews with the representative gynecologists every 2 months will provide us with this information. Process evaluations at the end of the study will be based on a professional questionnaire, a questionnaire for the patients’ representatives, and an addendum to the patients’ questionnaire in the after measurement.

## Discussion

The study is expected to yield important new evidence about the effects of a multifaceted approach on the improvement of patient-centeredness, patients’ QoL and levels of distress in fertility care. Determinants at patient and clinic levels of a change in these variables will also be assessed. By having knowledge of these results, patient-centered care and thus quality of healthcare can be improved. This may lead to a higher patients’ QoL, lower levels of distress in infertile couples, a reduction in patient discontinuing treatment prematurely and a reduction in healthcare costs.

To the best of our knowledge, this is the first study examining the effects of a multifaceted approach on patient-centered fertility care. In Dutch intensive care, a randomized trial is ongoing to determine the effect of a multifaceted approach on patient outcome and organizational process measures of care [57]. Completed studies examining the effects of a multifaceted approach on guideline implementation showed incompatible results [26,42,58]. In a systematic review on this subject the effects of different elements of a multifaceted approach were described, showing that the EOV is one of the most common evaluated interventions, resulting in modest improvements (6%, range −4 to 17.4%) in process of care [40]. Audit and feedback and patient-directed interventions appeared to result in modest (7.0%, range 1.3 to 16.0%) and moderate (20.8%, range 10.0 to 25.4%) effects, respectively [40]. Lewin and colleagues evaluated the effects of different interventions to promote patient-centered care [9]. Significant effects on patient satisfaction were demonstrated when using multifaceted approaches instead of usual care [59,60]. The majority of these studies were undertaken in the area of primary care. In fertility care, no overall sustainable effect of a multifaceted approach was found over audit and feedback on the level of guideline implementation [37]. This is in line with other studies on audit and feedback [39].

In sum, studies examining the effects of a multifaceted approach generally show slight improvements on patients’ well-being and patient-centered care. However, no clear evidence is available regarding how many and what combination of interventions provides the highest improvement in quality of care. One of the strengths of our study is that we will use a multifaceted approach consisting of three different interventions, which has been shown to be effective in different studies [39] and is based on known professional barriers [19,37,38]. Further, our outcome measures will be determined by validated and internationally developed questionnaires enhancing our study results. Finally, because one-third of all Dutch hospitals from all regions in our country will be approached for participation, representativeness of Dutch infertile couples can be ensured. Owing to these strong elements of our study, our results provide more evidence about the effectiveness of a multifaceted approach on patient-centeredness, patients’ QoL and levels of distress in fertility care.

## Trial status

Between June 2011 and October 2011, 32 clinics have been found willing to participate in this study. At the time of submission of the manuscript, the execution of the multifaceted approach, aiming at improving patient-centered fertility care in the 16 intervention clinics, has just started.

## Abbreviations

EOV: educational outreach visit; IVF: *in vitro* fertilization; QoL: quality of life.

## Competing interests

The authors declare that they have no competing interests.

## Authors’ contributions

AGH, RPMGH, CV, JAMK and WLDMN designed this study and were involved in developing the protocol. AGH drafted the manuscript. NTLvD and WLDMN revised critically for important intellectual content. All authors read and improved the final manuscript.
